# A feasible method to evaluate fetal palate: sequential sector-scan through oral fissure

**DOI:** 10.1186/s12884-023-05450-7

**Published:** 2023-03-09

**Authors:** Xiaofeng Lu, Yi Zhou, Min Fan, Wanyan Li, Chaoxue Zhang

**Affiliations:** 1 Department of Ultrasound, The First Affiliated Hospital of Anhui Medical University, No.218 Jixi Road, Shushan District, Hefei, Anhui China; 2 Dermatology, The First Affiliated Hospital of Anhui Medical University, Hefei, Anhui China

**Keywords:** Cleft palate, Cleft lip, Ultrasound, Prenatal diagnosis

## Abstract

**Background:**

Prenatal diagnosis of cleft palate is still challenging. To describe a practical and efficient method, sequential sector-scan through oral fissure (SSTOF), to evaluate palate.

**Methods:**

According to the characteristics of the fetal oral anatomy and ultrasonic directivity, we designed a practical method, sequential sector-scan through oral fissure, to evaluate the fetal palate, and the approach was verified efficiently by following up results of those fetuses with orofacial cleft who were induced because of their accompanied lethal malformations. Then, the 7098 fetuses were evaluated using sequential sector-scan through oral fissure. Fetuses were followed up after birth or induction to validate and analyze prenatal diagnosis.

**Result:**

According to the scanning design, sequential sector-scan through oral fissure was performed successfully from the soft palate to the upper alveolar ridge in induced labor fetuses, and the structures were displayed clearly. Among 7098 fetuses, satisfactory images were obtained for 6885 fetuses and the remaining 213 fetuses’ images were unsatisfactory because of fetuses’ positions and pregnant women with high BMI. Among 6885 fetuses, 31cases were diagnosed CLP or CP, which were confirmed after delivery or termination. There were no missing cases.

**Conclusions:**

SSTOF is a practical and efficient method to diagnose cleft palate, which might be applied to evaluate the fetal palate in prenatal diagnosis.

**Supplementary Information:**

The online version contains supplementary material available at 10.1186/s12884-023-05450-7.

## Background

Orofacial clefts are one of most common congenital defects which include cleft palate. Cleft palate is generally categorized as two types, cleft lip-palate (CLP) and isolated cleft palate (CP) [1], both of them may occur alone or accompany other anomalies, and some of cleft palate have certain correlation with other congenital defects and genes [2]. Cleft palate can lead to many long-lasting difficulties in speech, hearing, swallowing and mid-face protrusion. According to severity of the disease, the difficulty of surgery and repair are different. Beyond doubt, the disease will pose a burden to individuals, families and society [3]. However, the Eurofetus (European multi-center study) reported detection rates of 22%and 1.4% for CLP and CP [4]. As an important tool for prenatal diagnosis, ultrasound has been recognized as the major protocol for diagnosing cleft palate. However, due to the low display rate of fetal palate, the guidelines do not require palatal examination. The purpose of this study is to describe a feasible method “sequential sector-scan through oral fissure” to screen fetal palate. It might be very useful to improve the palate display rate and detect cleft palate.

## Methods

**Fig. 1:**
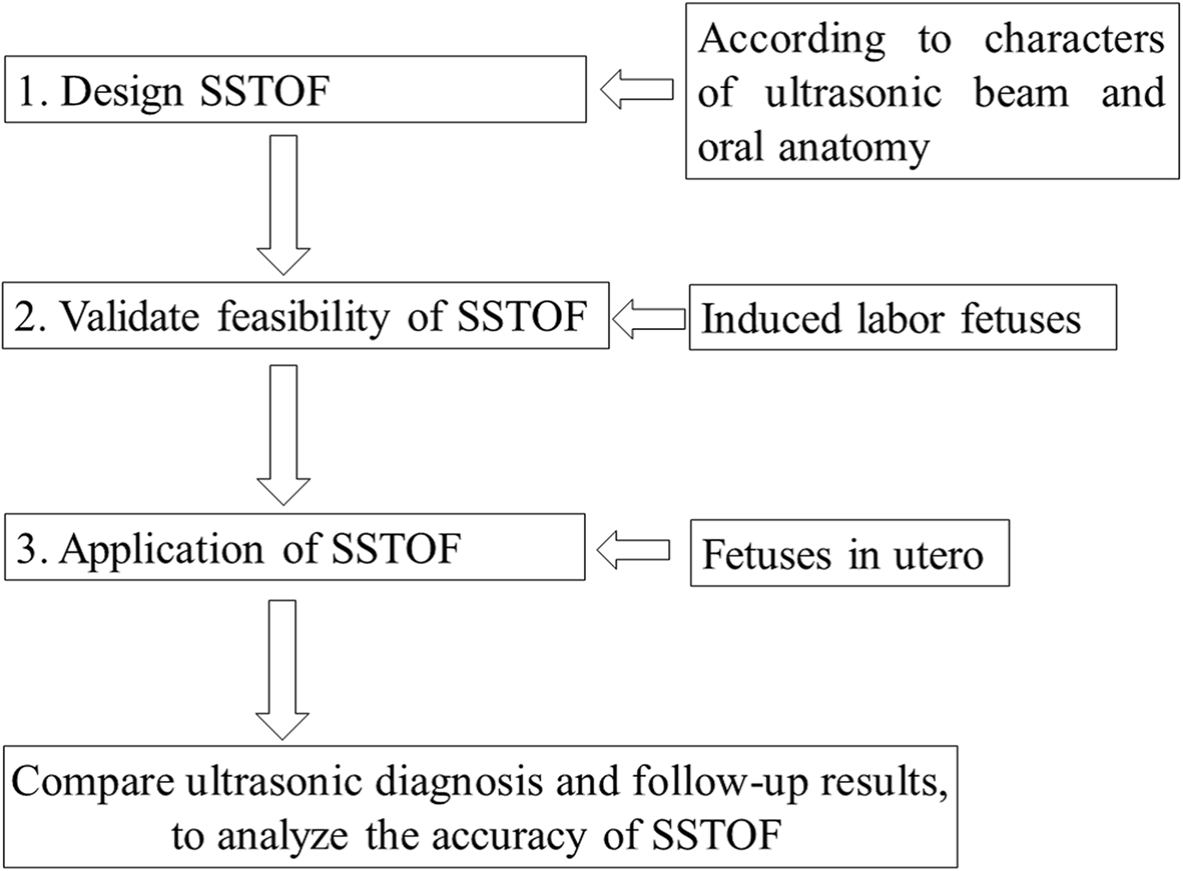
Experimental flowing chart

The process of research is shown in Fig. 1. All pregnancies signed informed consent. The study was conducted in accordance with the World Medical Association Declaration of Helsinki and approved by the Ethics Committee of the First Affiliated Hospital of Anhui Medical University.

Ultrasound Equipment: Mindray M7 Portable Color Doppler machine (probe:5-14 MHz), GE Voluson8 and Voluson10Color Doppler machine (proble:1-6 MHz). The sonographer who has been practicing prenatal diagnosis for at least 5 years.

### Design of scanning method of “sequential sector-scan through oral fissure”

**Fig. 2:**
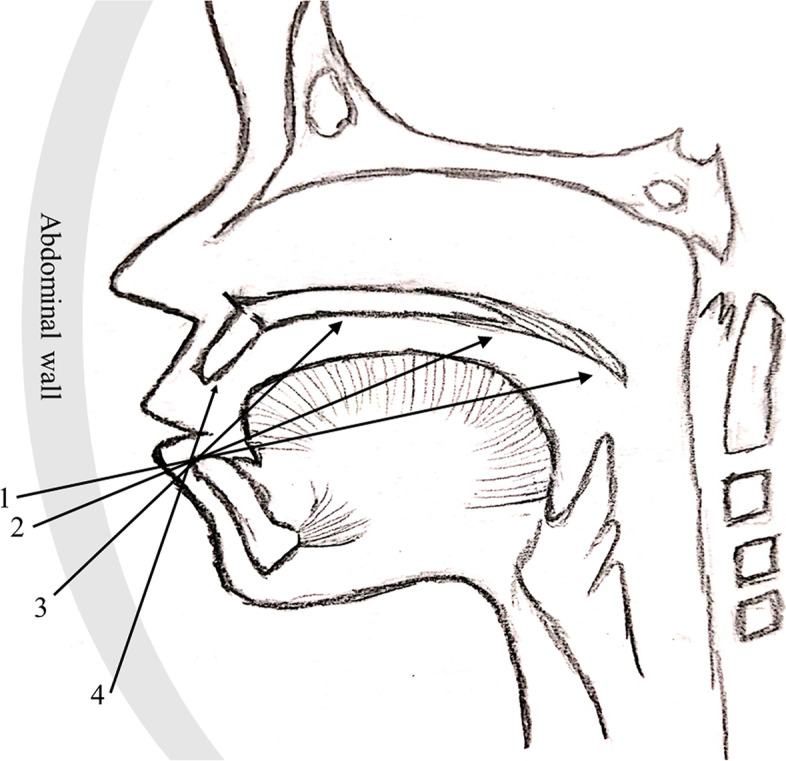
Scanning method design of SSTOF. 1/2/3/4 respectively represent continuous sequence sections. The evaluation was based on the dynamic scanning video, and the still picture was only the schematic diagram of the four anatomical marks captured in the vide

It has been reported that the scanning of fetal hard palate was completed by the axial transverse views method, but this method has high requirements on fetal position and only focuses on the hard palate. In order to more easily display the complete structure of the palate [5], based on the characteristics of the ultrasonic beam and the fetal oral anatomy, we designed “sequential sector-scan through oral fissure” scanning method (Fig. 2) [6]. First, adjust the direction of the beam according to the position of the fetal, so that the beam is directly in front of the fetal face, If the fetal position is poor, the sound beam can be adjusted to the side front. The acoustic beam was placed on the superior margin of the submaxilla parallel to the lower alveolar ridge plane through the oral fissure (Fig. 2, cross-Sect. 1). Then, the probe that pivoted from the superior margin of the submaxilla slightly tilted to head side, and soft palate (Fig. 2: cross-Sect. 2), hard palate (Fig. 2: cross-Sect. 3) and upper alveolar ridge (Fig. 2: cross-section of 4)will be displayed in sequence. The integrity of the palate was observed by dynamic sequential sector scanning.

### Verify feasibility of “sequential sector-scan through oral fissure

First, we verified the feasibility of sequential sector-scan through oral fissure with induced labor fetuses: Gross specimens of 5 cases induced labor (gestational age 25–27 weeks), the reasons for induced labor were serious congenital diseases or lethal malformations rather than orofacial clefts.

Second, Prenatal ultrasound guidelines indicate that the optimal time for fetal anatomical examination is between 18 and 24 weeks, but recommend that the second structural examination be performed in late pregnancy between 28 and 32 weeks. After 32 weeks, the fetus should be larger, relatively less amniotic fluid, and no fetal structural examination should be performed. In order to verify the feasibility of the method, we included 18–32 weeks into the validation range. From May to October 2020, 7098 fetuses (gestational age 18–32 weeks, mean: 24 ± 1.6 weeks)underwent prenatal screening by using the sequential sector-scan through oral fissure, and the ultrasonic videos and images were stored. The full-term fetuses and the induced labor fetuses were assessed by related specialized clinical doctors after birth. All fetuses were followed up by dialing phone and checking the inpatient medical records. The display rate of the palate and the diagnostic accuracy of cleft palate were analyzed.

## Result

**Fig. 3:**
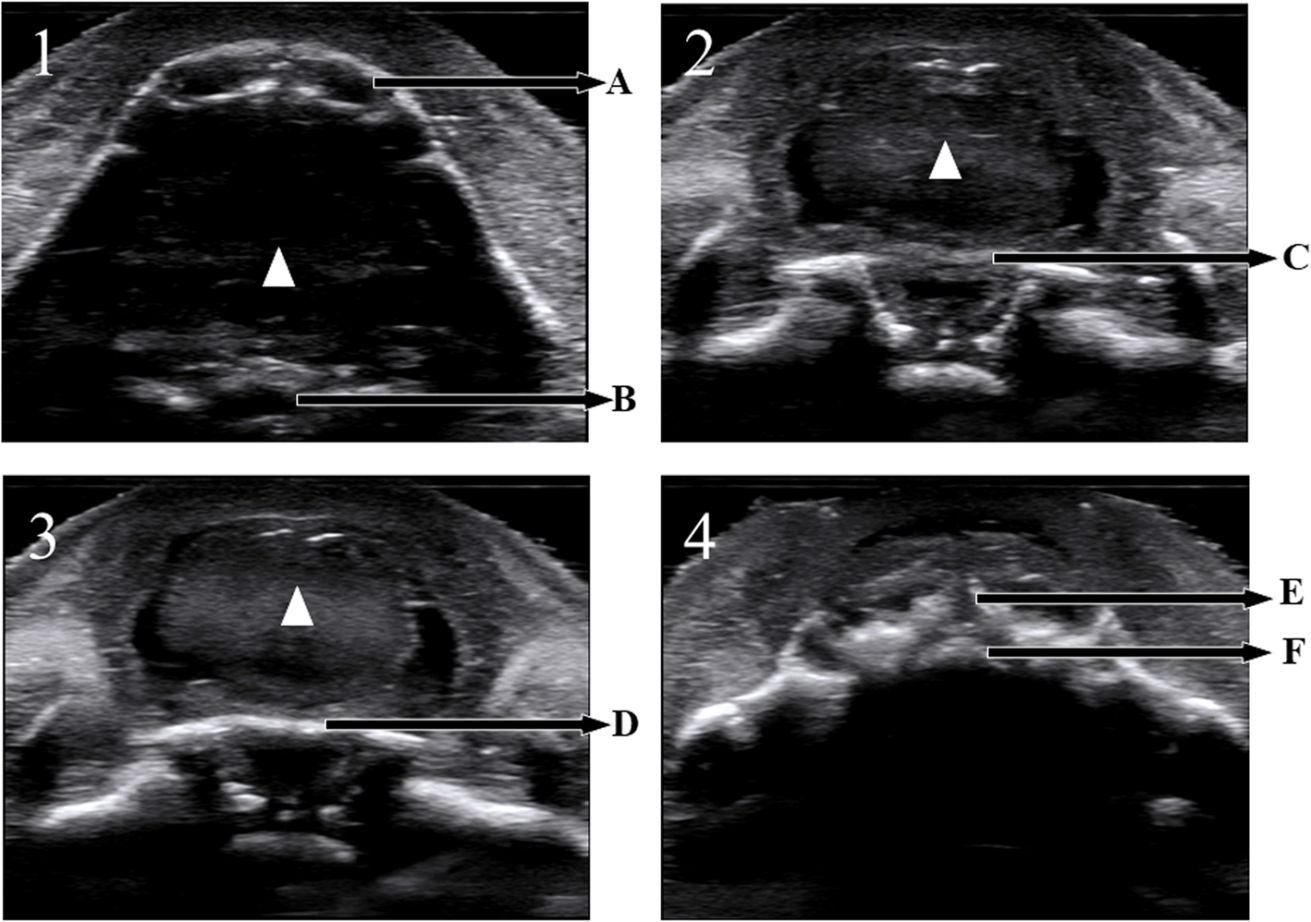
Verify the feasibility of SSTOF with induced labor fetus (gestational age:18 W). 1/2/3/4 respectively represent continuous sequence sections. **A**: lower alveolar ridge. **B**: pharynx. **C**: soft palate. **D**: hard palate. **E**: upper alveolar ridge. **F**: Primary palate. The white triangle: tongue

For in induced fetuses, sequential sector-scan through oral fissure was successfully performed to display the soft palate to upper alveolar ridge (Fig. 3, video1). The soft palate, hard palate and upper alveolar ridge can be displayed clearly in sequence. Lower alveolar ridge (Fig. 3A) and pharynx (Fig. 3B) were displayed. The normal soft palate displayed as continuous low echo (Fig. 3: C), the normal hard palate displayed a continuous high echo (Fig. 3: D), and the normal upper alveolar ridge displayed as high and low echoes of the arrangement rules (Fig. 3: E).

**Fig. 4:**
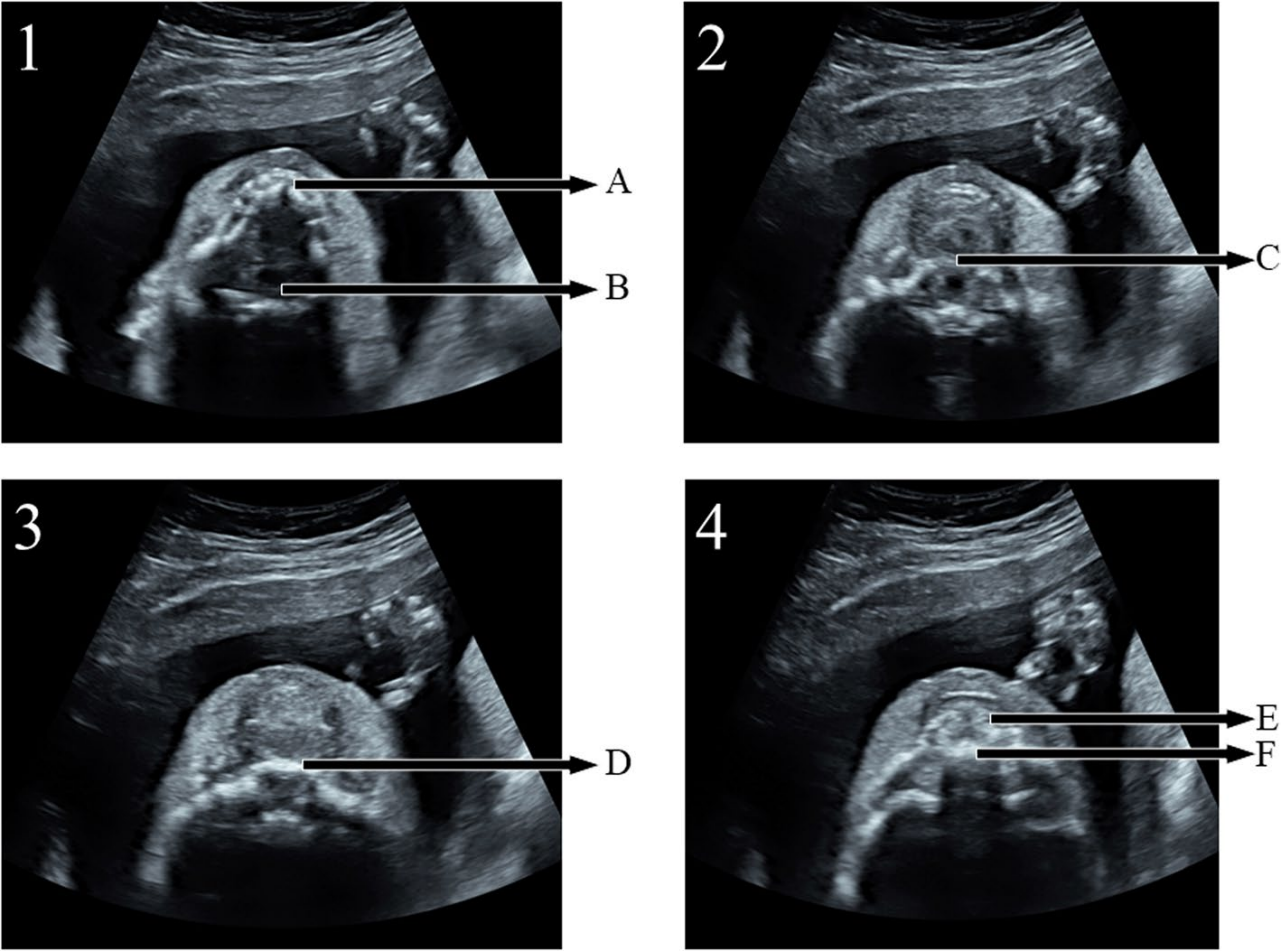
Applied SSTOF on the routine examination (from the front of fetal face, gestational age:23 W). 1/2/3/4 respectively represent continuous sequence sections. **A**: lower alveolar ridge. **B**: pharynx. **C**: soft palate. **D**: hard palate. **E**: upper alveolar ridge. **F**: Primary palate

**Fig. 5:**
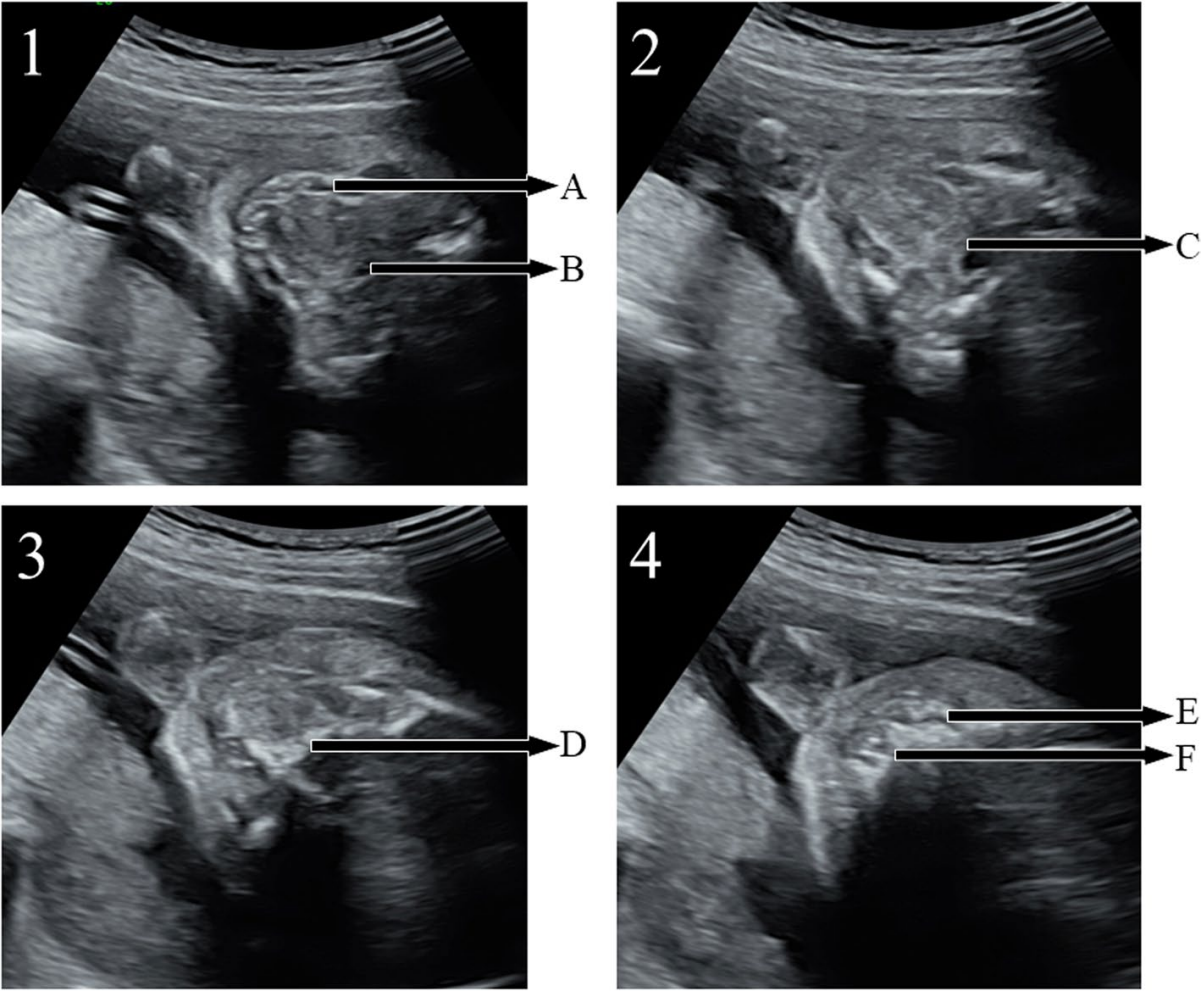
Applied SSTOF on the routine examination (from the side of fetal face, gestational age:22 W). 1/2/3/4 respectively represent continuous sequence sections. **A**: lower alveolar ridge. **B**: pharynx. **C**: soft palate. **D**: hard palate. **E**: upper alveolar ridge. **F**: Primary palate

7098 fetuses were collected in the study, for 213 fetuses, satisfactory images were not obtained due to some factors, include: excessive fetal dorsiflexion, pregnant women with high BMI and so on. The palatal display rate was 97% (6885/7098) in examination by using sequential sector-scan through oral fissure. The palatal structure can be clearly displayed, whatever the direction of ultrasonic beam is from the front or the side of the fetal face (Fig. 4 and video2, Fig. 5 and video3).

**Fig. 6:**
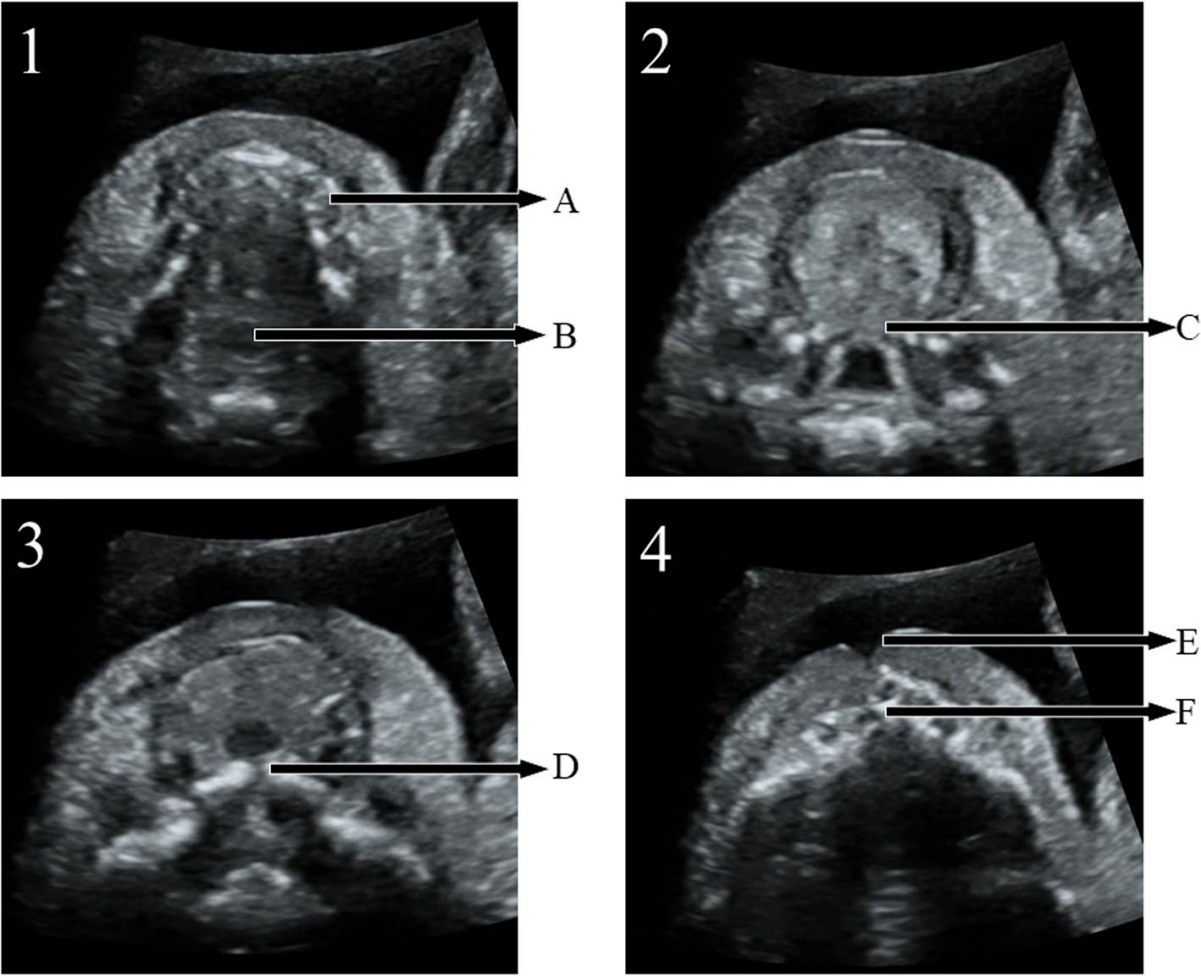
The case of CLP (gestational age:22 W). 1/2/3/4 respectively represent continuous sequence sections. **A**: lower alveolar ridge. **B**: pharynx. **C**: soft palate. **D**: cleft hard palate. **E**: cleft lip. **F**: cleft upper alveolar ridge

Among the 6885 fetuses, 31 cases were diagnosed as abnormal palatal development, including 8 cases of bilateral CLP, 17 cases of unilateral CLP, and 6 cases of isolated CP; In addition, 2 cases of bilateral CLP, both hard and soft palates were broken, and the other cases were cleft hart palate; Of the 31 cases, 28 were first detected in our hospital. Another three fetuses with CP at 28 to 32 weeks were referred to our center due to maternal complications during pregnancy. All the 6885 fetuses were followed up and no missed cases were found, the detection rates of CLP and CP were 100%.(Fig. 6,video4).

In this study, 16 of the 31 abnormal cases were accompanied by other system abnormalities, the most common was cardiac system, follow by nervous system. Invasive chromosome examination was performed for 7 cases of multiple malformations, including 2 cases of trisomy syndrome, 2 cases of 22q11.2 microdeletion syndrome, and 3 cases with no obvious abnormality.

## Discussion

CLP is not a fatal abnormality, but it may be accompanied by multiple abnormalities of other systems, Other systems should be carefully examined when the CLP is found, and the development of the lip and palate should also be observed when other systems are found. In addition, when CLP or CP is accompanied by other system abnormalities, it is recommended to carry out Invasive chromosome testing to provide guidance for the outcome of this pregnancy and the next pregnancy. Therefore, it is very important to make a clear diagnosis of CLP or CP before delivery.

Defects of the lip and upper alveolar ridge can be diagnosed successfully relative easily by conventional two-dimensional (2D) ultrasound, but the diagnosis of abnormalities of the palate remains a challenge. It is difficult to obtain a clear image of posterior or secondary palate due to its dome-shaped structure and acoustic shadow results from the upper alveolar ridge and maxilla. Only when the fetus head is totally extended, can we observe fluid between the fetal tongue and the palate. Three-dimensional (3D) ultrasound has been reported to improve the display of the fetal palate. However, as a screening tool 3D technology is time-consuming and requires specific skills of operators. The quality of 3D imaging can be also affected by fetal position and amniotic fluid factors. Therefore, the display rate of fetal palate is low and no guidelines has mentioned it. Currently, indirect methods for evaluating the palate, including overlapping lines were observed on the mid-sagittal plane in the second trimester [7], the retronasal triangle (RT) [8, 9] and maxillary gap (MG) [10] in the first trimester, are also available, but missed diagnosis still occur, RT cannot be used to diagnosed single secondary cleft palate [11]. MG has value only for the median cleft palate and it is difficult to display the non-median cleft [12]. The equal sign is used to diagnose cleft of soft palate in 20–25 weeks of gestation [13]. Florent FUCHS et al. proposed the axial transverse view of fetal face method, but this method is time-consuming and can only observe the hard palate [5].

There is lack of approach that can display the complete palate. We designed an approach according to the characteristics of ultrasonic beams and oral anatomy- “sequential sector-scan through oral fissure”.

Theoretically, the superior margin of submaxilla was used as a fulcrum, and the fan scan was performed to tilt from the pharynx to the upper alveolar ridge consecutively, so the soft palate, hard palate and upper alveolar ridge were displayed in sequence. In this procedure, the ultrasonic beam passes through fissure rather than forming teeth. Therefore, the image quality of fetal palate can hard be affected by the shape of the palate and shadow.

This method has been verified efficiently by induced labor fetuses. We also applied method to a large sample and obtained acquisition rate of 97% of fetal palate display. Except the cases that the fetal face was in position of hyperflexion. the whole view of the palate could be observed in all other positions by adjusting the acoustic beam, and there was no missed diagnosis or misdiagnosis. In addition, to verify the operability of the method, the proficiency level of the examiner has little influence. We have designed an ultrasound training program to train doctors’ screening for fetal palate, and the results revealed that 20 doctors, who had no experience in prenatal diagnosis of fetuses, were trained to perform palate scans skillfully. This further confirms the feasibility of our approach [14].

The shortcoming of the study is that the fetus is small and difficult to operate in the first trimester. In the trimester, the sensitivity for major defects is not more than 50% [15, 16]. Diagnosis of orofacial cleft, apart from severe cases, is not straightforward at this stage and therefore false-positive cases might have an important impact on couples. in terms of anxiety until the second-trimester ultrasound, when diagnosis can be definitively made. In addition, the technique requires suitable amniotic fluid in fetuses. With fetuses’ growth and the volume of amniotic fluid decrease, the views of SSTOF increasingly became difficult. So only a few fetal palates can be observed clearly in third trimester.

## Conclusion

The sequential sector-scan through the oral fissure can clearly display palate. The novel approach may be helpful for evaluating cleft palate.

## Supplementary Information

**Additional file 1:** **Video 1.** Obtaining the palate in Induced Labors, It visualized from lower alveolar ridge to hard plate by SSTOF clearly and dynamically.

**Additional file 2:** **Video 2.** Obtaining the palate in normal fetus from the front of fetal face. It visualized from lower alveolar ridge to hard plate by SSTOF clearly and dynamically.

**Additional file 3:** **Video 3.** Obtaining the palate in normal fetus from side of fetal face. It visualized from submaxilla to hard plate by SSTOF.

**Additional file 4:** **Video 4.** Cases of CLP. The echo of hard palate, lip and upper alveolar ridge interrupt.

## Data Availability

All data generated or analyzed during this study are included in this article. Further enquiries can be directed to the corresponding author.

## Abbreviations

2D: 2-dimensional
3D: 3-dimensional
CLP: Cleft lip-palate
CP: Isolated cleft palate
US: Ultrasound
SSTOF: Sequential sector-scan through the oral fissure
